# Combined Presigmoid-Subtemporal Approach in a Semi-Sitting Position for Petroclival Meningiomas: A Technical Report

**DOI:** 10.7759/cureus.19609

**Published:** 2021-11-15

**Authors:** Daniel S Leon-Ariza, Rubby J Romero Chaparro, Lisa Rosen, Juan S Leon-Ariza, Fidias E Leon-Sarmiento, Juan Villalonga, Alfredo Quinones-Hinojosa, Alvaro Campero

**Affiliations:** 1 Radiation Oncology, Miami Cancer Institute, Miami, USA; 2 Neurological Surgery, Hospital Dr. Raúl F. Larcade, Buenos Aires, ARG; 3 Neuroscience, Mediciencias Research Group, Miami, USA; 4 Environmental Health, Florida International University, Miami, USA; 5 Internal Medicine, Universidad Nacional de Colombia, Bogota, COL; 6 Neuroscience, Miami Neuroscience Institute, Baptist Health South Florida, Miami, USA; 7 Neurological Surgery, Universidad Nacional De Tucumán, San Miguel de Tucumán, ARG; 8 Neurological Surgery, Mayo Clinic College of Medicine and Science, Jacksonville, USA; 9 Neurological Surgery, Hospital Ángel C. Padilla, San Miguel de Tucumán, ARG

**Keywords:** petroclival meningiomas, semicircular canals, presigmoid approach, skull base, cranial nerve

## Abstract

The removal of petroclival meningiomas (PMs) is considered a neurosurgical challenge due to the critical mobilization of key neurovascular structures. Limited knowledge about the benefits of operating on patients with PMs using the combined presigmoid-subtemporal approach (CPSA) in a semi-sitting position has precluded its generalizability. We report on ten patients with PMs operated in a semi-sitting position using CPSA. We remark that before the surgical approach was accomplished in our group of patients, the CPSA via semi-sitting position was conducted and standardized in six adult cadaveric heads. The neuroanatomic dissections made in cadavers allowed us to confidently use CPSA in our set of patients. There were no comorbidities, perioperative complications, or deaths associated with the surgical procedure. CPSA via a semi-sitting position can be considered a safe approach to remove PMs.

## Introduction

Petroclival meningiomas (PMs) are considered difficult-to-access tumors because of their deep location within the brain. Further, the intimate relation of these tumors with critical surrounding neurovascular structures makes resection of PMs one of the most challenging procedures in neurosurgery. PMs excision has been done using some surgical approaches with miscellaneous outcomes [1-3]. The most common approaches reported at present are retrosigmoid, pre-sigmoid, transpetrosal, Hakuba’s anterior petrosectomy, transotical, orbitozygomatic, combined supratentorial, and infratentorial, and a combined orbitozygomatic and posterior fossa approach [4]. A combined presigmoid-subtemporal approach (CPSA) while operating the patient in a semi-sitting position has been informed previously in limited case reports precluding its generalizability [4,5].

Here, we report the largest sample of patients operated to date with PMs in semi-sitting position using a novel CPSA. Our objective was two-fold: First, to determine the anatomical landmarks of the CPSA in cadaveric heads; second, to examine the safety of this approach in patients with PMs operated in a semi-sitting position. Our results indicate that PMs can be successfully and safely removed using this positioning and approach, and they may help to re-orient current practices.

## Technical report

Cadaveric study

**Figure 1:**
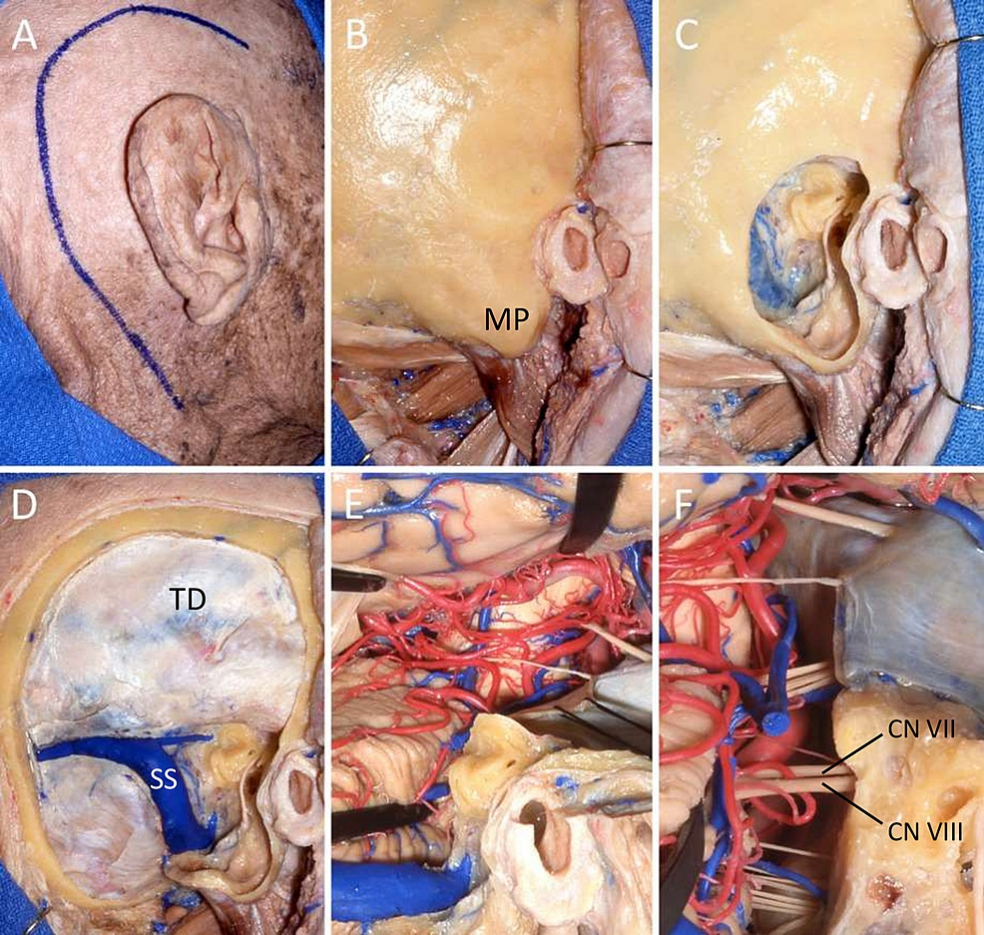
CPSA on cadaveric model A: Initial incision following ear borders (3 cm above and 3 cm behind). B: Exposure of the mastoid process, the temporal bone, and the squamous part of the occipital bone. C: Drilling of the mastoid process exposing the sigmoid sinus behind, the facial nerve and the semicircular canals on the front, below the jugular vein bulb, and the temporal dura mater above. D: The approach is completed with a combined subtemporal and presigmoid craniotomy. E: View of the approach once the dura mater is opened and the tentorium is resected from lateral to medial. F: Magnified view of the approach. The semicircular canals were drilled. CN VII: cranial nerve VII; CN VIII: cranial nerve VIII; CPSA: combined presigmoid-subtemporal approach; MP: mastoid process; SS: sigmoid sinus; TD: temporal dura

Six adult cadaveric heads, fixed in formaldehyde and injected with colored silicone were studied. Using the CPSA, we assessed the flexibility of cranial regions involved in PMs, the visibility of adjacent nerve and vascular structures, as well as the surrounding anatomy to simulate the surgical removal of PMs. The CPSA was bilaterally done in all cadaveric specimens by neurosurgeons imitating the surgical procedure to be further done in a set of patients (Figure 1).

Results (Cadaveric Dissection)

Excellent visualization of the petroclival area was documented in all cadaveric specimens. All nerve and blood vessels surrounding the region of interest were clearly visualized and prepared for the surgical resection. We successfully reached the petroclival region through a CPSA without complications. We concluded that the CPSA enables a novel route to excise PMs, and offers enough flexibility for tumor removal with few, or none, extra neurovascular tissue manipulation. The technique used in the cadavers was replicated in our group of patients and a detailed description follows.

Surgical technique

Surgical Plan

We performed two procedures on each patient. Initially, we determined the surgical approach, and 48 hours later, we resected the tumor. In cases where the vestibulocochlear cranial nerve (CN VIII) was affected by the tumor, a trans-labyrinthine approach was considered. Conversely, if the CN VIII was intact, we performed a retro-labyrinthine approach. Perioperative complications including those associated to operate patients in a semi-sitting position such as tension pneumocephalus, macroglossia, peripheral nerve damage, quadriplegia, and venous air embolism were also assessed [4,6,7].

Patient Positioning

**Figure 2:**
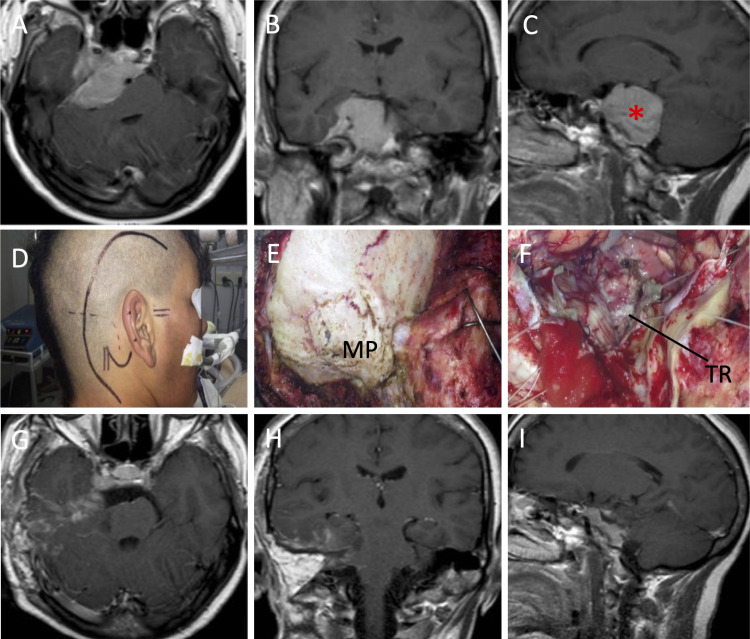
Surgical application of CPSA in a semi-sitting position A-C: Preoperative MRI, D: Incision, E: Bone exposure, F: View after tumor resection, G-I: Postoperative MRI. Asterisk: petroclival meningioma (sagital view). CPSA: combined presigmoid-subtemporal approach; MP: mastoid process; TR: tumor removal

The patient is positioned in a semi-sitting position, which can be achieved in a stepwise manner as described elsewhere [8]. In brief, the operating table is gradually bent and tilted to obtain a trunk-to-inferior-limbs right angle and a 30° knee flexion. Then, the upper platform of the operating table was manually unblocked and bent to 20°-30° to diminish the need for head flexion. The arms are then properly positioned. The Mayfield head holder is then fixed and the head rotated 10º towards the lesion. Further, a 3-4 cm space between the mandible and the sternum is maintained to guarantee a normal neck venous outflow. Once the correct final patient positioning was achieved, skin markings and a horseshoe-shaped incision were made (Figures 1A, 2D). Figure 2 also shows the preoperative (A-C) and postoperative (G-I) MRIs.

Soft Tissue Dissection

Preserving the temporal fascia and muscle is critical to avoid vascularization issues during the reconstruction phase. Therefore, the removal of the myocutaneous flap is performed in a posterior to antero-inferior direction, following a caudal course to the zygoma. After this, the sternocleidomastoid muscle is removed from the mastoid process in the direction of the external auditory canal and the temporal bone avoiding thermal damage to the CN VII. Once the dissection of the soft tissue is accomplished, hook retraction and elastic bands are applied [9].

Craniotomy

Careful head rotation is completed as mentioned (see Patient Positioning section). Burr holes are made above the lateral end of the transverse sinus, and then lateral to the transverse-sigmoid junction [10]. The superior semicircular canal (SC) and the posterior SC are resected on top from the lateral SC bone posteriorly to the anterior wall of the sigmoid sinus. This traces an imaginary area limited by the Donaldson line that helps to increase the exposure area of the petroclival region [11]. The mastoidectomy is completed and the cortical bone surrounding SCs is exposed. Then, the tympanic portion of the CN VII and lateral canal located deep to the spine of Henle is exposed. Trautmann’s triangle, an area limited by the jugular bulb inferiorly, the middle fossa dura superiorly, and the superior petrosal sinus and sigmoid sinus posteriorly is exposed; then, the dura is opened anterior to the sigmoid sinus. Afterward, the superior petrosal sinus is ligated, the tentorium is opened from lateral to medial, and the presigmoid dura is opened anteriorly to the sigmoid sinus. Opening of the temporal dura and division of the tentorium is made preserving the vein of Labbé. For the retro-labyrinthine presigmoid exposure, preservation of the SCs should be considered. Exposure of the CN VIII is performed medially in relation to the CN VI and CN VII. Then, downward retraction of the CN VIII exposes the nervus intermedius and the CN VII. Further, a diamond burr skeletonizes the CN VII. SCs and vestibule removal are completed for the trans-labyrinthine approach. Later, the cochlea is removed for completion of the transcondylar exposure extending to the lateral clivus and the inferior petrosal sinus.

For neurovascular protection, a three-step process is followed. First, anterior exposure of the vertical region of the petrosal portion of the ipsilateral carotid artery is done in relation to the jugular bulb; second, the lateral segment of the basilar artery and anterior pons deeply located to the exposure are identified; third, the superior cerebellar artery, which passes below the CN IV and CN V is carefully manipulated. Hemodynamic aspects during brain manipulation should also be monitored aiming to prevent triggering a type I, II, or III trigeminocardiac reflex [12,13].

Tumor Exposure and Resection

Once the durotomy and the neurovascular protection steps are accomplished, the CPSA is achieved, and the exposure is completed (Figure 1). The arachnoid layer located above the tumor is opened and the mass is debulked. After tumor removal, bone drilling should be considered when tumor extension is at the level of the internal auditory meatus and the jugular foramen. Therefore, exposure and removal of the tumor should follow a piece-by-piece resection (Figure 2F).

Reconstruction

A multilayer closure reconstruction includes the fat, fascia, and flap; they are carefully re-implanted to prevent major cerebrospinal fluid (CSF) fistulae. Then, the flap is sealed with fibrin glue, and the reconstruction phase is completed.

Surgical outcomes

We operated on 10 patients. One of them had a prior tumor resection using a different approach. Female to male ratio was 2:1. The mean age of presentation of the tumor was 54.5 ± 13 years. Headache was the main presenting symptom (50%). The most affected cranial nerve prior to the surgery was the CN VIII (70%). Critical vascular regions such as the cavernous sinus were affected by the tumor (60%). A retro-labyrinthine approach was completed in 30% of the patients who had CN VIII intact. Efforts were made to achieve a gross total resection of the tumor. Of the patients, 20% had transient CN deficits. A cerebrospinal fluid fistula was the most frequent complication (20%), which was managed by a triple flap., and sealed with fibrin glue. There were no permanent comorbidities, perioperative complications or deaths associated to the surgical procedure. Patient data are summarized in Table 1.

**Table 1:** Demographic, clinical, and surgical aspects of patients diagnosed with petroclival meningioma a: Transitional deficit, b: Permanent deficit, c: Lower cranial nerves. *: Tumor adhered to brainstem, **: Calcified tumor CN: cranial nerve; CSF: cerebrospinal fluid; F: female; LCN: lower cranial nerves; M: male; PM: petroclival meningioma

Case	Gender	Age (ys)	Clinical Findings	Cranial Nerve	Cavernous Sinus Extension	Grade of Resection	Complications (CN)
1	M	35	Cerebellar syndrome; Hemiparesis	VII, VIII	+	Sub Total*	III^a^
2	F	36	Cerebellar syndrome; Headache	VIII^c^	+	Gross Total	IV^b^
3	F	62	Headache	None	-	Gross Total	VIII^b^
4	F	55	Headache; Hemiparesis	V, VI, VIII	+	Gross Total	Multiple
5	F	72	Headache; Cerebellar syndrome	V	-	Gross Total	CSF Fistula
6	M	66	Cerebellar syndrome	VIII	+	Sub Total**	None
7	F	49	Hemiparesis; Cerebellar syndrome	V, VI, VIII	+	Gross Total	III; VII^a^
8	F	42	Tetraparesis; Hydrocephalus	V, VII, VIII^c^	+	Gross Total	CSF Fistula
9	F	69	None	V	-	Gross Total	VII^a^
10	M	59	Headache; Cerebellar syndrome	VIII^c^	-	Gross Total	None

Quality of Life (QoL) assessment

QoL was determined by patient-generated responses to the Functional Assessment of Cancer Therapy questionnaire, including its brain and head and neck subscales. All patients had a better post-surgical QoL with respect to their neurological function, and 98% of them had an improved head and neck score. Independence was rarely affected, with Karnofsky Performance Scores of 90 to 100 in all patients.

## Discussion

The selection of surgical approach and patient positioning depends on the location, size, extension of the tumor, and preoperative imaging evaluation, among other aspects. We report for the first time the feasibility of operating patients with PMs in semi-sitting position using a CPSA. To our knowledge, this is the largest report of PMs patients undergoing this approach. Indeed, we searched in PubMed, Web of Science, and Scielo databases on similar topics; other than limited case reports, our search returned no entries for series of 10 or more PMs patients. Noteworthy, we had no deaths and neurovascular neurological complications due to the surgical procedure were very few with most of them being transitory. Our favorable outcomes could be due to several factors, including a well-rehearsed “surgical plan” based on the experience of neurosurgeons, and the neuroanatomic studies done beforehand in cadaveric specimens.

Careful neuroanatomical knowledge is mandatory to navigate successfully during surgeries, and mostly in cases involving manipulation of critical neural and vascular structures. Although online education is bringing another view to teach medicine, human cadaveric dissection is still the classical core tool to know and learn neuroanatomy [14,15]. Following this classical view, we clearly identified in our cadaveric material the neuroanatomical region to be operated on and addressed at that time potential complications derived from the planned surgical approach. Based on our findings, we favor the use of cadaveric specimens to improve neuroanatomy knowledge as well as master surgical dissection techniques aiming to excel not only PMs resection using a CSPA while patients are positioned in a semi-sitting position but also other neurosurgical procedures and approaches.

Compared to previously published approaches, the CPSA we accomplished allowed us to safely navigate through the complex surgical challenges originated by the location of PMs. CPSA has several advantages, such as surgical distance to the slope being shorter and visible, allowing the brainstem to be easily reached. Likewise, cranial nerves (e.g., CN III-CN VIII), basilar artery, and inner ear structures can be better protected [16-19]. Further, the venous sinuses including the sigmoid and transverse one as well as Labbé’s vein can also be preserved, and the blood vessels supplying the tumors can be better blocked at the initial stages of the surgeries increasing rates of tumor resection together with appropriate patient positioning.

Ideal patient positioning involves balancing surgeon comfort against risks to the patient having in mind the physiological changes and complications that may occur during a surgical procedure. We recommend semi-sitting position over the prone or supine position to remove PMs because of an easier anatomical orientation, safer surgical access to midline structures, and improvement for gravitational drainage of venous blood flow minimizing cerebrospinal fluid leakage; it also offers a better surgical exposure of deep brain areas such as the petroclival junction [5]. Further, this positioning allows a clean surgical field, reduces the need for bipolar coagulation, and facilitates cerebellar retraction. From an anesthetic standpoint too, a semi-sitting position has advantages such as better access to the patient's face to assess airways including endotracheal tube placement, and monitoring of cranial nerves; further, chest compression can be easier to perform in case of cardiac arrest [20].

Limitations

The main limitation of the present study is that there is no comparison with a similar cohort of patients harboring the same type of tumors and operated by the same medical team while patients are placed in other surgical positions. However, we did not find intraoperative semi-sitting position-related complications, so we consider that such a comparison would be at present non-significant. Conversely, we analyzed a homogeneous cohort of adult patients undergoing elective cranial surgery; thus, confounding factors such as brain and/or brainstem neurological emergencies or potential spine complications never happened. Another limitation could be the sample size; however, as aforementioned, this is the largest case series in patients with PMs successfully operated to date via CSPA in a semi-sitting position. Another limitation could be that we did not use intraoperative neurological monitoring, which is very useful to prevent unwanted neurosurgical outcomes. In our favor, we say that we took enough care during positioning of the patient as well as during the surgical interventions mastered ahead in cadaveric material allowing for successfully performing the planned surgical procedures without unwanted outcomes. However, intraoperative neurological monitoring is highly recommended in future studies dealing with PMs because of the critical neurovascular areas involved by these tumors. Lastly, although this is a single-center study, it was done with the same medical and nursing staff, using the same perioperative protocol guidelines; further, the same team was in charge of all patients. These factors together eliminate type II errors. Further, several physicians including senior and attending doctors, as well as trainees, were involved in patient positioning, surgical procedure, and postoperative management adding internal validity to our results by eliminating potential controlling bias. Therefore, we consider our technique could be generalized elsewhere using a similar surgical protocol.

## Conclusions

Our study indicates that the combination of technical factors described here is effective to safely remove PMs using CPSA via semi-sitting positioning. We emphasize dedicated pre-operative cadaveric disection to timely identify remedies to potential issues associated with the risk derived from the surgical approach and patient positioning introduced here. Replication of this technique in larger samples of participants is worth trying. Studies aimed to investigate clinical outcomes using this technique are highly encouraged.
